# Extralobar Pulmonary Sequestration: A Rare Entity

**DOI:** 10.7759/cureus.64977

**Published:** 2024-07-20

**Authors:** Anurag Rai, Shiva S, Vekhu Rhakho, Abhishek Choudhary, Shailendra Kumar

**Affiliations:** 1 Thoracic Surgery, King George's Medical University, Lucknow, IND; 2 General Surgery, King George's Medical University, Lucknow, IND

**Keywords:** bronchopulmonary sequestration, rare thoracic entities, thoracic surgery, ct angio, extralobar sequestration

## Abstract

Lung sequestration is a rare congenital anomaly characterized by non-functional lung tissue that lacks normal bronchial communication and receives blood supply from an aberrant systemic artery. Extralobar sequestration (ELS) is less common and usually found in the lower thoracic or upper abdominal regions. It is often diagnosed in infancy or early childhood due to associated congenital anomalies or respiratory symptoms. The complexity of highly variable anatomy and the involvement of multiple systemic arteries pose significant diagnostic and therapeutic challenges. Patients may present with a variety of nonspecific symptoms, such as recurrent respiratory infections, chronic coughs, or unexplained abdominal pain, which often leads to delayed diagnosis. This case report details the unique presentation of extrapulmonary lung sequestration in a 32-year-old male who presented with persistent respiratory symptoms and intermittent abdominal discomfort. Through comprehensive imaging studies and surgical intervention, the diagnosis was confirmed, and the sequestered tissues were successfully resected. This report aims to highlight the importance of considering bilateral extrapulmonary sequestration in differential diagnoses of recurrent respiratory and abdominal symptoms and to discuss the diagnostic approach and management strategies for this rare condition with highly variable anatomy.

## Introduction

Pulmonary sequestration (PS) is a rare lung anomaly and constitutes 0.15%-6.4% of congenital lung malformations, characterized by dysplastic pulmonary tissue mass with no connection with the tracheobronchial tree and supplied by an anomalous systemic artery. They are encountered in approximately 1%-2% of all lung resections [1]. Intralobar sequestration (ILS) and extralobar sequestration (ELS) are the two types described, with the former being more common and found in 75%-85% of cases [2]. Extralobar sequestration is most commonly found intrathoracically, usually on the left side, in the posterior costophrenic angle, with the mediastinum, pericardium, intra- or sub-diaphragmatic, and retroperitoneum as other sites of occurrence. The diagnosis can easily be missed in adults, as most of the symptoms and the imaging findings overlap with other more common pulmonary diseases, including lung cancer. This case underscores the need for high clinical suspicion and comprehensive imaging when diagnosing atypical thoracoabdominal symptoms and discusses the clinical features, diagnostic evaluation, surgical management, and outcomes of this rare condition.

## Case presentation

**Figure 1:**
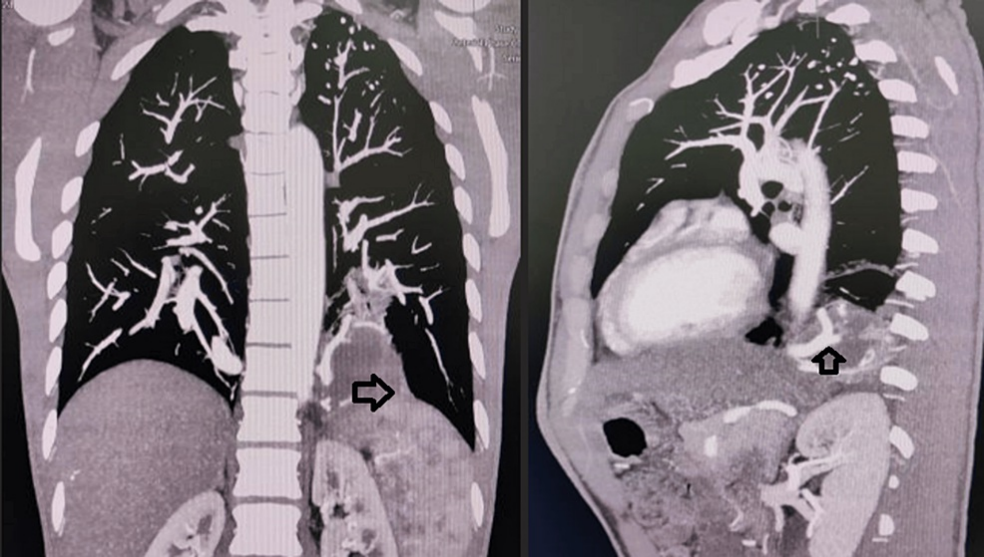
The coronal section of the CT angiogram shows the blood supply of sequestration tissue (arrow).

**Figure 2:**
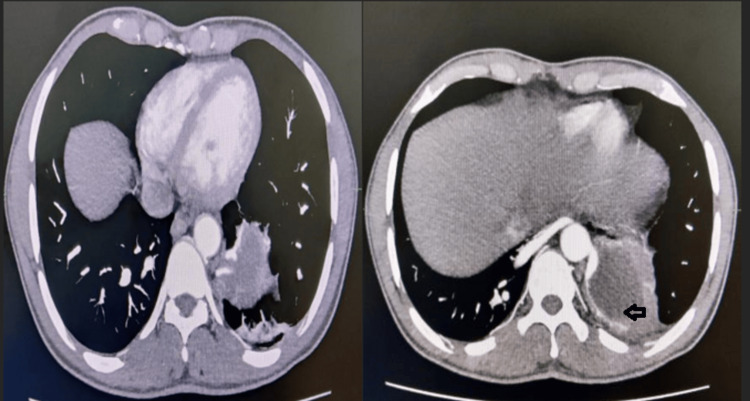
The axial section of the CT angiogram shows the sequestration tissue (arrow).

A 32-year-old male presented with complaints of non-radiating left-sided chest pain associated with intermittent fever, productive cough, dyspnea on exertion, and hemoptysis for three months. He presented to a local hospital, where a high-resolution computed tomography (CT) of the thorax was done, which was suggestive of a left-sided loculated pleural effusion, and a left-sided intercostal chest tube drainage tube (ICD) was inserted along with intravenous analgesics and antibiotics. As there was no alleviation of symptoms, he was then referred to us. On presentation, the patient was tachypneic, requiring low-flow oxygen. His respiratory system examination revealed reduced air entry in the left lower lung field and bronchovesicular sounds in the right lung field. His blood counts, renal function tests, liver function tests, arterial blood gas analysis, erythrocyte sedimentation rate (ESR), C-reactive protein (CRP), chest X-ray, and electrocardiogram were normal. The chest scan was suggestive of left lung basal consolidation and right hilum consolidation. A repeat CT scan of the thorax was done, which was suggestive of left chest wall pleural mass measuring 9.31 mm x 9.88 cm between the 9^th^ and 11^th^ ribs, with mild pleural thickening on the left side, subpleural fibrotic streaks in bilateral lower lobes, and consolidation in the right lower lobe. A flexible bronchoscopy was done, which suggested a normal appearance of the bronchopleural tree. The bronchioalveolar lavage fluid examination was negative for tuberculosis. A CT pulmonary angiography showed a well-defined soft tissue density lesion measuring 53.4 x 63.2 x 45.5 mm with internal hypodensity in the posterobasal segment of the left lower lobe, showing no communication with the bronchial tree or pulmonary arteries, no evidence of visceral pleura surrounding the mass, and being supplied by one of the branches of the descending thoracic aorta at D9-D10 vertebral level, which was suggestive of intrapulmonary, extra-lobar sequestration (Figures 1, 2).

**Figure 3:**
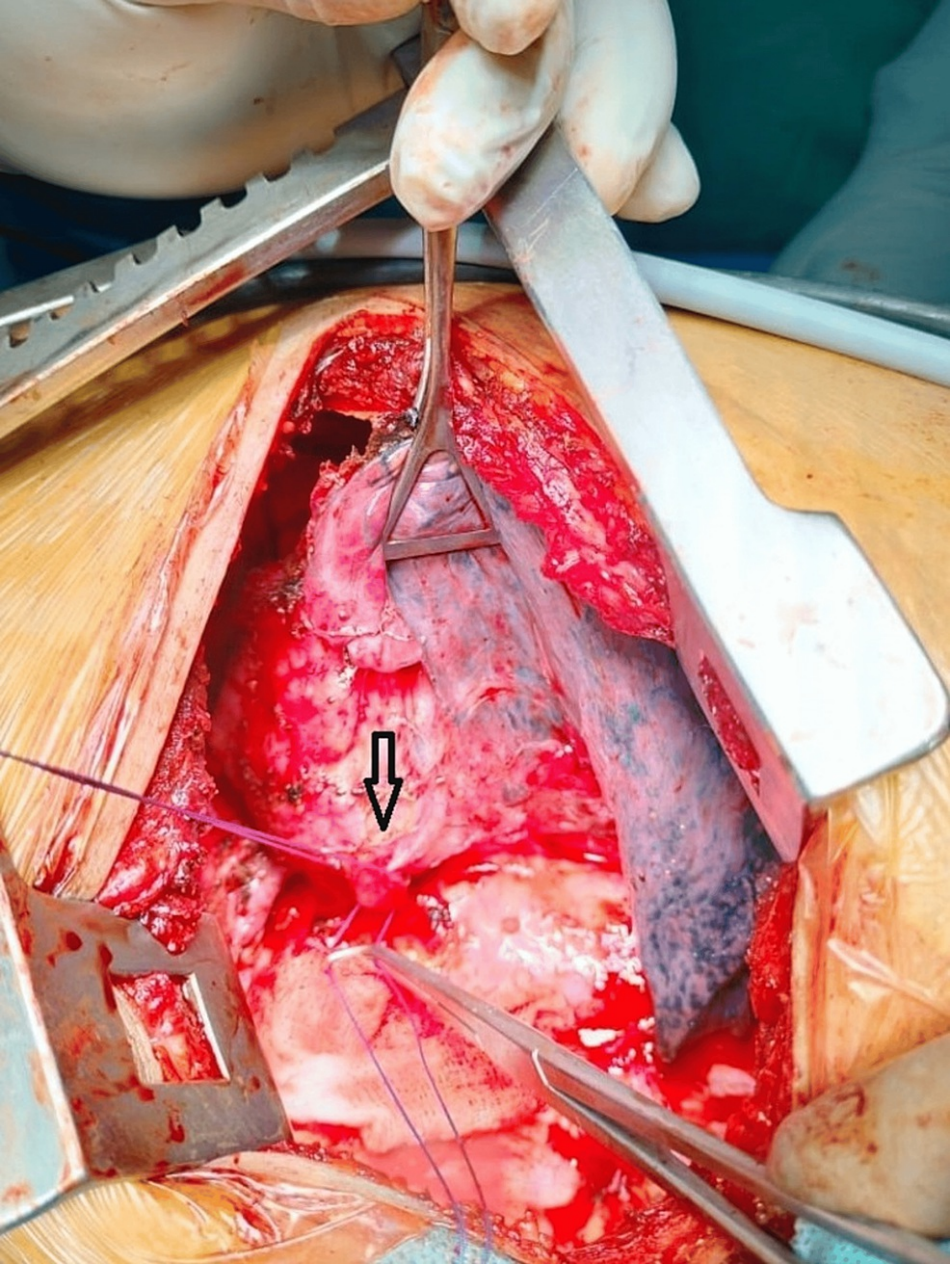
Intraoperative sequestrated tissue along with two transfixed arterial supplies from the thoracic aorta (arrow)

A biopsy of the mass showed fibrocollagenous tissue enclosing a few glands lined by cuboidal to columnar cells with surrounding dense lymphomononuclear cell infiltrate and no evidence of granuloma or malignancy. The patient was taken up for a left-sided thoracotomy. Intraoperatively, a cystic mass of size 5 x 5 cm was found adhered to the left lower lobe of the lung with a maintained plane, no parenchymal communication, and deriving its blood supply from the thoracic aorta (Figure 3), suggesting ELS and excision was done.

The intraoperative and postoperative periods were uneventful. The patient’s condition improved after surgery, and he was discharged in stable condition.

## Discussion

Pulmonary sequestration is a rare congenital disorder, accounting for less than 6% of all reported congenital lung abnormalities [2]. It consists of aberrant and non-functional pulmonary tissue that is not connected to the normal bronchopulmonary system and is fed by systemic vascular supply, usually originating at the level of the thoracic aorta [3]. Extralobar sequestration, which is presented in this case, is separate from the normal lung, has its own pleural covering, and is most commonly found intrathoracically, with only 10%-15% occurring below the diaphragm.

In 60%-65% of cases, ELS is associated with other congenital anomalies, including congenital diaphragmatic hernia, diaphragmatic eventration, foregut duplication, congenital cystic adenomatoid malformation, bronchogenic cyst, and rarely cardiovascular, genitourinary, and gastrointestinal anomalies, but none of them could be found in the index case [4,5]. Sequestrations are also included in the spectrum of broncho-pulmonary-foregut malformations, as they may communicate with the esophagus or stomach. Usually, the original communication with the foregut regresses, but occasionally it may persist as a patent tract or as an obliterated fibrous cord [6]. Extralobar sequestrations usually (80%) derive their blood supply directly from the thoracic or abdominal aorta, as seen in the index case; rarely (15%) they may derive their blood supply from gastric, splenic, intercostal, and subclavian arteries; and very rarely (5%) they may derive their blood supply from both systemic and pulmonary circulation [1]. The usual venous drainage is into the right atrium via systemic veins, with rare drainage into suprarenal, portal, intercostal, and other abdominal veins, and very rarely into the left atrium through the pulmonary veins [7].

Although the pathophysiology of ELS is unclear, the accepted hypothesis is the non-absorption of internal organ capillaries, which initially branch to the dorsal aorta around the primitive alimentary cavity and lung bud, leading to abnormal aortic branches of blood vessels responsible for the formation of lung isolation due to the dissemination of the embryonic lung tissue [4].

The clinical features of the two types of sequestration are different. Extralobar sequestration, which is usually diagnosed on prenatal USG, may be asymptomatic or might present with respiratory failure due to lung hypoplasia or compression of lung parenchyma [2]. Extralobar sequestrations rarely get infected, as their pleural attachment prevents contact with inhaled air. In adult patients, hemothorax, lung infarction, infection, hemoptysis, and chest pain are the usual clinical manifestations, as seen in the index case [4]. In contrast, ILS usually manifests during childhood, and about half of them present after 20 years. 

The gold standard modality of choice for diagnosis is a CT scan with contrast. There are cases of ELS being diagnosed during routine pregnancy ultrasound and Doppler studies [8]. The use of invasive angiography is becoming less common. The radiological differentials of intrabdominal sequestration include suprarenal neuroblastoma, teratoma, foregut duplication, adrenal hemorrhage, and mesoblastic nephroma.

The usual management is surgical excision, as there is a high rate of synchronous malformations such as congenital cystic adenomatoid malformations, which possess the possibility of malignant transformation. Excision can be done via thoracotomy, thoracoscopy, or robotic routes [9,10]. The safety and efficacy of the thoracoscopic route are well established without any increase in mortality or morbidity. In both routes, constant vigilance during surgery for aberrant vessels is crucial given the high rates of anomalous arterial vessels. Intralobar sequestration usually requires a lobectomy, while ELS can be managed by resection. Coil embolization and radiofrequency embolization are being used lately and have shown promising results with the regression of sequestration tissue within a few months [11,12].

## Conclusions

In conclusion, this case report highlights the rare occurrence of bilateral extrapulmonary lung sequestration, a congenital anomaly that poses significant diagnostic and therapeutic challenges. The patient’s presentation with recurrent respiratory infections and intermittent abdominal pain underscores the necessity of considering lung sequestration in the differential diagnosis of such symptoms. Advanced imaging modalities, such as CT and MRI, play a crucial role in accurately diagnosing this condition by identifying the aberrant arterial supply and the sequestered lung tissue. Surgical resection remains the definitive treatment for extrapulmonary sequestration, with the potential for excellent outcomes when appropriately managed. This case emphasizes the importance of thorough diagnostic evaluation and timely surgical intervention in managing extrapulmonary lung sequestration.
